# Knowledge and Attitudes About HIV/AIDS Among Adolescent Students in 15-19 Years Age Group Based on the Adolescent Education Programme: A Cross-Sectional Study Conducted in Kamrup (Metro), Assam

**DOI:** 10.7759/cureus.62122

**Published:** 2024-06-11

**Authors:** Ashfia Habib, Kumaril Goswami, Jutika Ojah, Mintu Dewri Bharali

**Affiliations:** 1 Department of Community Medicine, Gauhati Medical College and Hospital, Guwahati, IND

**Keywords:** adolescent education programme, attitude, knowledge, hiv/aids, adolescents

## Abstract

Introduction: There has been a rising trend in human immunodeficiency virus (HIV) cases globally as well as regionally. To combat the rising cases, it is very crucial to understand the perception of knowledge and attitude among the adolescent age group, as they constitute a majority of the population in India. The adolescent age group is quite vulnerable; hence, special attention has to be given to this particular age group to prevent them from acquiring high-risk behaviours. The Government of India initiated the Adolescent Education Programme to help adolescents cultivate a positive attitude and learn life skills to live a better and dignified life. Educational institutions have a significant role in imparting effective sex education to adolescents. This programme focuses on making the students aware of the dangers, stigma, and discrimination associated with HIV/acquired immunodeficiency syndrome (AIDS) as well as modes of transmission of HIV/AIDS, helping them to acquire the necessary life skills to enable them to avoid risky situations and to develop healthy and responsible behaviour. The study aims to assess the knowledge and attitude about HIV/AIDS among adolescent students aged 15-19 years and help them achieve a healthy attitude and responsible behaviour towards HIV/AIDS.

Methods: A cross-sectional study was conducted in the educational institutions of Kamrup (Metro), Assam, catering to the adolescent age group of 15-19 years for two months, beginning on March 15, 2024, and ending on May 15, 2024. Simple random sampling was done to select seven schools, and students were selected via systematic random sampling. The knowledge and attitude about HIV were assessed using a self-designed questionnaire that was pilot-tested; Cronbach’s alpha was used to check the internal consistency of the questionnaire; and content validation was done by a group of four experts on the subject matter. The survey questionnaire was administered to the participants in their classrooms after obtaining prior permission from the school authorities. Fisher’s exact test and Chi-square tests were done to test the associations, taking a p-value <0.05 as statistically significant. A binary logistic regression model was put up to show the influence of certain socio-demographic variables on knowledge and attitude about HIV/AIDS, taking a p-value <0.05 as statistically significant. A two-factor analysis of variance with measurement repetition was also performed.

Results: The majority, 165 (52.5%) of the participants, had good knowledge and attitudes regarding HIV/AIDS. The socio-demographic variables such as age, institution, grade, and religion were significantly associated with knowledge and attitude about HIV/AIDS (p-value <0.05). Regarding sources of knowledge about HIV/AIDS, the majority, 178 (56.7%) of the participants, mentioned it being taught in school or college as a part of the curriculum.

Conclusion: It is critical to address the current lack of information and unfavourable attitudes regarding HIV/AIDS among adolescents through school-based health programmes. This should be accomplished through proactive campaigns by educators, community leaders, non-governmental organizations (NGOs), and other groups in partnership with the government.

## Introduction

The Human Immunodeficiency Virus (HIV) has emerged as a major public health problem globally. Globally, the most vulnerable group of individuals to HIV infection is reported to be the youth, with adolescents contributing significantly [1]. India has the world's largest adolescent population, 253 million, with every fifth person aged 10 to 19 years old [2]. As per the Joint United Nations Programme on HIV/AIDS (UNAIDS) statistics, 39 million people globally were living with HIV in 2022, and 1.3 million people became newly infected with HIV in 2022. A total of 630,000 people died from AIDS-related illnesses worldwide, compared to 2.0 million people in 2004 and 1.3 million people in 2010 [3]. According to a UNICEF (2021) report, in 2016, male and female adolescents aged 15-19 years in India had very little complete and correct information about HIV/AIDS, with 28.20% and 18.50%, respectively [4,5]. According to the India HIV Estimates 2021, the National AIDS Control Organisation (NACO), and the ICMR-National Institute of Medical Statistics (2022), the HIV prevalence in the adult population (15-49 years) was predicted to be 0.21% in 2021. Adult HIV prevalence was highest in North Eastern states (2.70% in Mizoram, 1.36% in Nagaland, and 1.05% in Manipur), followed by southern states (0.67% in Andhra Pradesh, 0.47% in Telangana, and 0.46% in Karnataka). The total number of people living with HIV (PLHIV) was expected to be around 24 lakh. According to 2021 projections, the overall number of individuals living with HIV/AIDS in Assam was 25,073 [6]. According to the National Family Health Survey (NFHS) 5 (2019-2021), one-fifth of women and roughly one-third of males aged 15-49 years in India have extensive knowledge about HIV/AIDS [7]. The statistics are even lower for Assam when it comes to comprehensive knowledge about HIV/AIDS among the adult population aged 15-49 years; it was estimated at 19.2% in females and 25.3% in males [8].

Assam is classified as a low HIV prevalence state, with an estimated adult HIV/AIDS prevalence of 0.09%, less than the national average of 0.21%. According to the National AIDS Control Programme (NACO) estimation report for 2020, Assam has an anticipated 21,240 individuals living with HIV/AIDS (PLHA) in 2020. Though Assam is classified as a low HIV prevalence state, it is also extremely vulnerable to HIV transmission for a variety of reasons, including its location as a gateway to North Eastern states and its proximity to four high HIV prevalence states: Manipur, Mizoram, Nagaland, and Meghalaya. Another factor is that a huge number of young people migrate from Assam to larger cities for employment [9].

Taking statistics into account, the number of HIV cases in the North Eastern states increased significantly, posing a huge issue in the public health sector. The government of India has implemented an Adolescent Education Programme that is guided by the National Curriculum Framework (NCF), 2005, which recommends that “education should instill independence of thought and action, sensitivity to others’ well-being and feelings, learning to respond to new situations in a flexible and creative manner, a predisposition towards participation in democratic processes, and the ability to work towards and contribute to economic processes and social change” [10].

The Adolescent Education Programme features a special module highlighting the understanding of the HIV/AIDS epidemic and empowering adolescents to conduct informed and responsible behaviour. The module has distinct objectives for secondary and senior school pupils [10].

One of the most important tactics used in the prevention and control of HIV/AIDS around the world is the spread of HIV/AIDS information and awareness. Inadequate awareness and high-risk behaviour are important obstacles to avoiding the spread of HIV/AIDS. Peer pressure, misunderstandings about sexuality, the influence of mass media, and greater internet access predispose youngsters to pornographic images that thrill them, and finally, they engage in hazardous sexual practices, therefore predisposing them to HIV/AIDS infection [1]. There have been numerous national and international studies on the knowledge and attitude regarding HIV/AIDS, but there are currently no published articles that address the effects of incorporating the Adolescent Education Programme in the National Curriculum Framework, 2005, on the knowledge and attitude about HIV/AIDS among the adolescent age group.

This study takes into consideration both the knowledge and attitude towards HIV/AIDS among the adolescent age group as well as an overview of the effectiveness of the Adolescent Education Programme. The aim of this study was to assess the knowledge about HIV/AIDS and attitudes towards HIV/AIDS-infected individuals among the adolescent students in Kamrup (Metro) using a questionnaire based on the Adolescent Education Programme included in the National Curriculum Framework, 2005, as well as the source of knowledge about HIV/AIDS.

## Materials and methods

The study was an analytical cross-sectional study done in the educational institutions of Kamrup (Metro). It was completed during a two-month period, beginning on March 15, 2024, and ending on May 15, 2024. The study's participants were adolescent students aged 15 to 19 years, currently studying in Kamrup (Metro). The sample size was computed using data from the most recent NFHS 5. According to the NFHS 5 (2019-2021) statistics, the estimated proportion of comprehensive knowledge among Indian adults aged 15-49 years was 25.80% [11]. The sample size was determined using the Cochran formula: 

 n = design effect * (1.96)^2^pq/ d^2^ = 310

where p = 0.26, q = 1-p = 0.74, d (relative error) = 6%, design effect: 1.45 (intraclass correlation coefficient was taken as 0.005).

**Figure 1:**
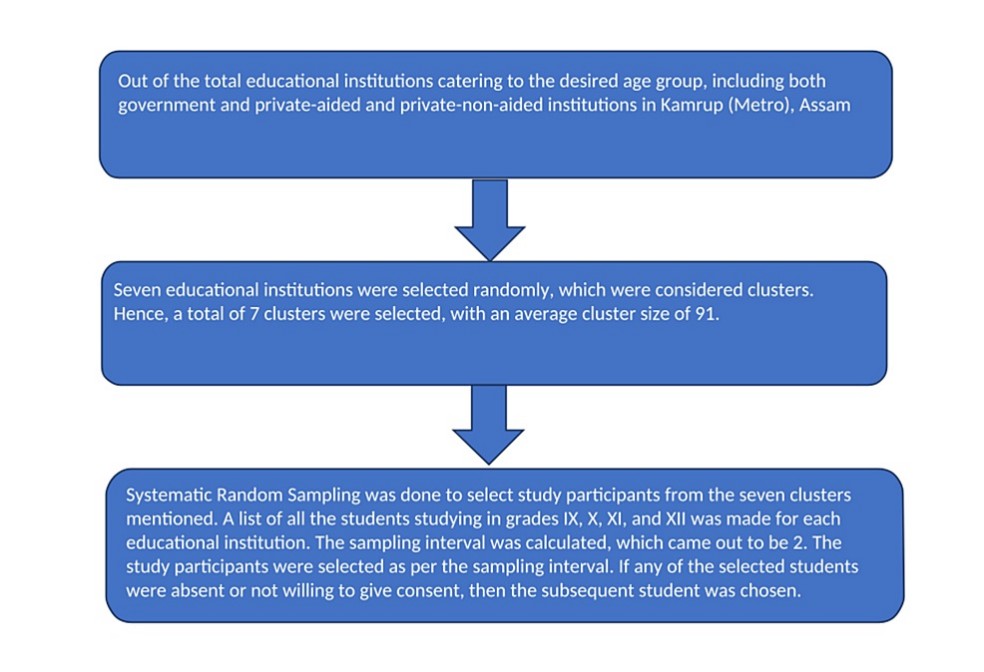
Flowchart for the sampling technique of the study

Taking a non-response rate of 1%, the sample size is calculated as 310 + 1% of 310, which comes out to be 313.1; a sample size of 314 was taken into consideration. The educational institutions were selected using simple random sampling; seven institutions were selected out of the total educational institutions catering to the desired age group, and the study participants were selected using systematic random sampling. The flow chart for the same is mentioned below (Figure 1).

The study participants were assessed based on a self-designed questionnaire that was pilot tested among adolescents in the age group of 15 to 19 years, other than the 314 study participants.The questionnaire was validated via a content validation approach, as four experts familiar with HIV/AIDS were consulted to approve the eight questions about knowledge and attitude and their scale. Reliability was tested using Cronbach’s α coefficient (total Cronbach’s α was found to be 0.84). 

Adolescents (15 to 19 years old) who gave consent were included in the study, and those who were absent on the day of the study or who were not willing to give consent were excluded. The data collection tool was a self-designed questionnaire that was pre-tested and structured to assess the knowledge and attitude about HIV/AIDS among adolescent students aged 15-19 years. The questionnaire consisted of five questions regarding knowledge about HIV/AIDS and three questions regarding attitude towards the disease. These questions were based on the Adolescent Education Programme launched in 2005 by the Ministry of Human Resource Development (MHRD). There were three parts to the questionnaire. The first part had details of the socio-demographic data as well as sources of knowledge regarding HIV/AIDS; the second and third parts had questions regarding knowledge and attitudes towards HIV/AIDS. The questionnaires provided were both in English and in the local language (Assamese). The translation from English to the Assamese version of the questionnaire was done by an expert. Ethical clearance was obtained from the Institutional Ethics Committee at Gauhati Medical College and Hospital (ECR/1470/Inst/AS/2020). Verbal consent was obtained from the study participants. An assurance of confidentiality was provided to the participants. The data were collected, later coded, and entered into an IBM-compatible computer using SPSS software, version 25.0 (IBM Corp., Armonk, NY). The data were presented in the form of tables and figures. Descriptive statistics were done for the socio-demographic variables. Fisher’s exact test and Chi-square tests were done to test the associations, taking a p-value <0.05 as statistically significant. A binary logistic regression model was put up to show the influence of certain socio-demographic variables on knowledge and attitude about HIV/AIDS, taking a p-value <0.05 as statistically significant.

## Results

A total of 314 adolescent students, both boys and girls, from seven different schools located in Kamrup (Metro) were enrolled in this study. Out of the total 314 students, the majority, 190 (60.5%), were male. The majority, 246 (78.4%) of the study participants, belonged to the age group of 17 years and below. The mean age of the participants was 16.35 ± 1.22 years. The majority, 118 (38%) of the study participants, belonged to grade X, and the mean of the grade was 10.29±1.06. Out of the total participants, 232 (73.9%) study participants were from English-medium institutions. Out of the total participants, 201 (64%) study participants were from non-public institutions. The majority, 172 (55%) of the study participants, were Hindus (Table 1).

**Table 1:** The frequency and percentage of the various socio-demographic variables The majority 190 (60.5%) of the study participants belonged to the gender male, the majority 246 (78.3%) belonged to the age group 17 years and below, and the majority 118 (38%) were from grade X. English was the medium for the majority of 232 (73.9%) of the study participants. The majority 201 (64%) of the study participants were from public institutions, and the majority 172 (55%) were Hindu by religion.

VARIABLES		FREQUENCY (n)	PERCENTAGE (%)
Gender	Male	190	60.5
	Female	124	39.5
Age	≤17	246	78.3
	>17	68	21.7
Grade	IX	83	26
	X	118	38
	X+	113	36
Medium	English	232	73.9
	Others	82	26.1
Institution	Public	201	64
	Private and Others	113	36
Religion	Hinduism	172	55
	Islam	84	27
	Christian	41	13
	Others	17	5

The socio-demographic variable “occupation” was classified as per ISCO 08 (International Standard Classification of Occupation 08) (Table 2).

**Table 2:** The frequency and percentage of the occupation of parents as per ISCO 08 The majority, 184 (58.60%) of the study participants, mentioned “professionals” as the occupation of the father, and the majority, 233 (74.20%) of the study participants, mentioned that their mothers were housewives, followed by other professions. ISCO 08: International Standard Classification of Occupation 08

Variables		Frequency (n)	Percentage (%)
Occupation of Father	Professionals	184	58.60
	Technicians and Associate Professionals	56	17.83
	Services and Sales Workers	31	9.87
	Craft and related Trades Workers	20	6.37
	Armed Forces	8	2.55
	Skilled, Agricultural, Forestry and Fishery Workers	6	1.91
	Clerical Support Workers	6	1.91
	Plant and Machine Operators and Assemblers	2	0.64
	Unemployed	1	0.32
Occupation of Mother	Homemaker	233	74.20
	Professionals	46	14.65
	Craft and related Trades Workers	18	5.73
	Services and Sales Workers	14	4.46
	Technicians and Associate Professionals	3	0.96

**Figure 2:**
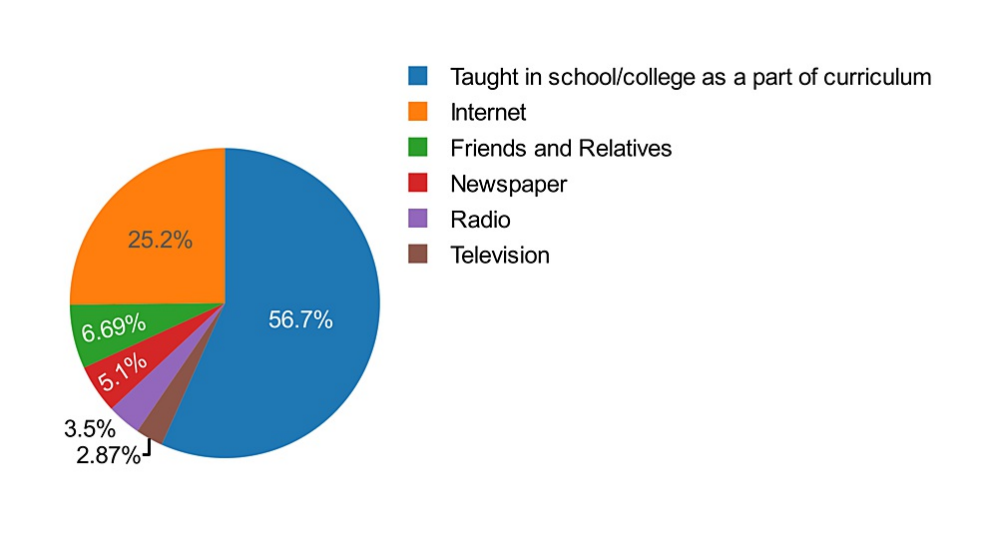
A pie chart showing the source of knowledge about HIV/AIDS The pie chart here gives an idea regarding the fact that the majority, 178 (56.7%) of the study participants, knew about HIV/AIDS as it was taught in their educational institutions, followed by the internet, friends, and relatives. The majority mentioned it being taught in school or college as a part of the curriculum.

Regarding sources of knowledge about HIV/AIDS, the majority, 178 (56.7%) of the participants, mentioned it being taught in school or college as a part of the curriculum (Figure 2).

The questions about knowledge and attitude were given scores of 0, 1, and 2 as per the available options. Later, when the answers given by each participant were summed up, a minimum score of 0 and a maximum score of 16 were taken into account. The sum of the scores was further classified into 3 categories: poor knowledge and attitude (score ranging from 0 to 5), average knowledge and attitude (score ranging from 6 to 11), and good knowledge and attitude (score ranging from 12 and above). The majority, 165 (52.5%) of the participants, had good knowledge and attitudes regarding HIV/AIDS; 144 (45.9%) had average knowledge; and five (1.6%) had poor knowledge. The responses to the questions about HIV/AIDS are mentioned in Table 3. Out of the total study participants, 10 participants who scored the highest in the questions regarding knowledge performed poorly in the questions regarding attitude (scores for attitude ranged from 0 to 3).

**Table 3:** The distribution of the study sample as per the responses to the questions about knowledge and attitudes toward HIV/AIDS-infected persons The majority, 250 (79.6%) of the study participants, knew about the sexual mode of transmission of human immunodeficiency viruses/acquired immunodeficiency syndrome (HIV/AIDS); the majority, 202 (64.3%), knew about the mother-to-child transmission; the majority, 202 (64.3%), knew about the route of HIV/AIDS transmission through contaminated blood; the majority, 185 (58.9%), knew about the transmission through the usage of contaminated syringes; and the majority, 194 (61.8%), agreed that HIV/AIDS is not transmitted through touching, shaking hands, hugging, sharing utensils, coughing, and sneezing. The majority 192 (61.1%) of the study participants agreed against the prohibition of HIV/AIDS-infected people in schools and colleges; the majority 183 (58.3%) agreed against the isolation of HIV/AIDS-infected people; and the majority 189 (60.2%) agreed that indulging in activities like playing with an HIV/AIDS-infected person is acceptable.

Questions	Responses	Frequency (n)	Percentage (%)
Q.K1 Do you agree that a person can get infected with HIV by having unprotected sex with the infected partner?	Agree	250	79.6
	Don’t know	51	16.3
	Disagree	13	4.1
Q.K2 Do you agree that there is a risk of mother-to-child transmission if the mother is infected with HIV?	Agree	202	64.3
	Don’t know	69	22
	Disagree	43	13.7
Q.K3 Do you agree that we can get HIV infection if we get HIV-contaminated blood?	Agree	202	64.3
	Don’t know	55	17.5
	Disagree	57	18.2
Q.K4 Do you agree that using the same syringe with an HIV-infected person can transmit HIV?	Agree	185	58.9
	Don’t know	81	25.8
	Disagree	48	15.3
Q.K5 Do you agree that HIV is not transmitted through touching, shaking hands, hugging, sharing utensils, coughing, and sneezing?	Agree	194	61.8
	Don’t know	65	20.7
	Disagree	55	17.5
Q.K6 Do you agree that it is acceptable for an HIV-infected person to study in the same school as us?	Agree	192	61.1
	Don’t know	84	26.8
	Disagree	38	12.1
Q.K7 Do you agree that it is acceptable for an HIV-infected person to live freely without isolation?	Agree	183	58.3
	Don’t know	74	23.6
	Disagree	57	18.1
Q.K8 Do you agree that we can indulge in any activities like playing with an HIV-infected person?	Agree	189	60.2
	Don’t know	64	20.4
	Disagree	61	19.4

The age, institution, grade, and religion of the study participants were found to be significantly associated with their knowledge and attitude about HIV/AIDS (Table 4) on applying Fisher’s exact test and Chi-square test. A binary logistic regression was performed to examine the influence of only the significant variables like age, institution, grade, and religion on the variable “Category 2,” which is good knowledge and attitude. Logistic regression analysis showed that the model as a whole is significant (Chi ^2^(7) = 49.37, p <.001, n = 314). A significant association was found with the socio-demographic factors age (p-value <.003, 95% CI = 0.28-0.77), grade (p-value <.001, 95% CI = 2.12-6.71), and religion (p-value 0.004, 95% CI = 0.12-0.68). The adjusted odds ratio of 0.47 suggests a 53.23% decrease in the odds of the outcome for each additional unit of Age. The adjusted odds ratio of 3.8 suggests a 280.13% increase in the odds of the outcome for each additional unit of Grade. The adjusted odds ratio of 0.29 suggests a 70.97% decrease in the odds of the outcome for each additional unit of Religion Christian (Table 5).

**Table 4:** The association between socio-demographic variables and knowledge and attitude about HIV/AIDS The association between socio-demographic variables like age, institution, grade, and religion is found to be significantly associated with the level of knowledge and attitude. The p-value of <0.05 is taken as statistically significant.

Variables		Good Knowledge and Attitude	Poor and Average Knowledge and Attitude	Two-sided p-value
Age	≤17	111(35.4%)	135(43%)	<0.0001
	>17	54(17.2%)	14(4.4%)	
Institution	Public	96(31%)	105(33%)	0.0317
	Private and others	69(22%)	44(14%)	
Grade	IX	29(9.2%)	54(17.2%)	<0.0001
	X	61(19.4%)	57(18.2%)	
	X+	75(24%)	38(12%)	
Religion	Hinduism	57(18.2%)	27(8.5%)	0.0038
	Islam	86(27.4%)	86(27.4%)	
	Christian	15(4.8%)	26(8.3%)	
	Others	7(2.2%)	10(3.2%)	

**Table 5:** Binary logistic regression model showing the influence of socio-demographic variables on the level of knowledge and attitude regarding HIV/AIDS The logistic regression model presented here shows a significant influence of age, institution, grade, and religion on the dependent variable, which is Category 2 of the level of knowledge and attitude about HIV/AIDS. The model was derived by taking the reference category for institutions as private and the reference category for religion as Islam. Here, a p-value of <0.05 is taken as statistically significant. The goodness of fit measures from the logistic regression analysis: -2 Log-Likelihood (385.11), Cox & Snell R² (0.15), Nagelkerke R² (0.19), McFadden’s R² (0.11).

Variables	Coefficient B	Standard error	z	p	Adjusted Odds Ratio (OR)	95% conf. interval (CI)
Age	-0.76	0.25	2.99	.003	0.47	0.28 - 0.77
Institution Public	0.27	0.27	0.97	.33	1.31	0.76 - 2.24
Institution Others	-1.8	1.13	1.6	.11	0.16	0.02 - 1.51
Grade	1.34	0.3	4.49	< .001	3.8	2.12 - 6.81
Religion Hinduism	-0.56	0.3	1.85	.065	0.57	0.32 - 1.03
Religion Christian	-1.24	0.43	2.85	.004	0.29	0.12 - 0.68
Religion Others	-0.9	0.57	1.58	.115	0.41	0.13 - 1.25

**Figure 3:**
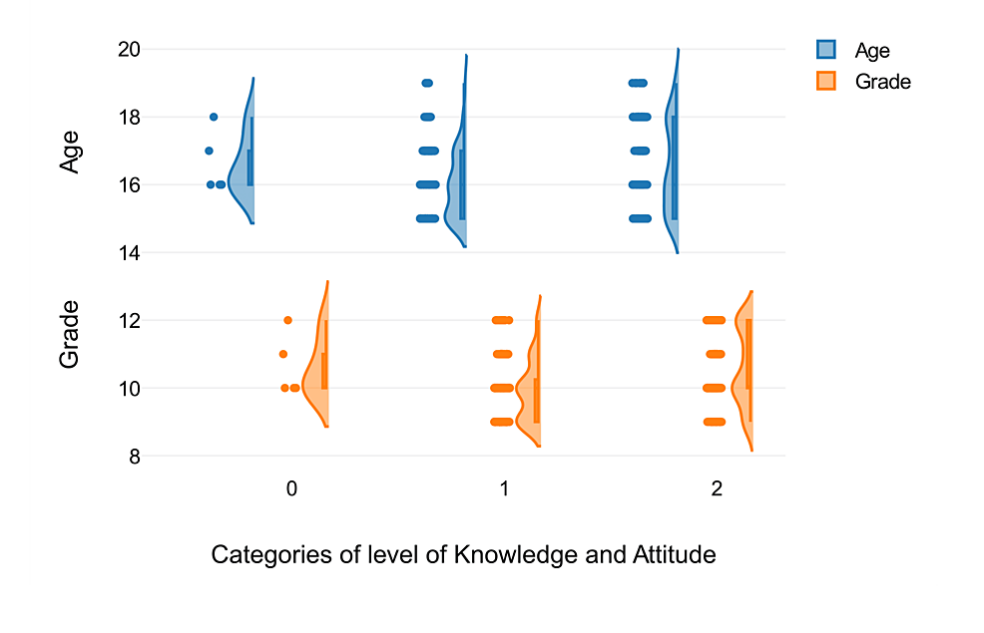
Raincloud plot showing the socio-demographic variables age and grade with respect to categories of level of knowledge and attitude The raincloud plot is given for effective visualisation; it indicates the data distribution (kernel density estimate) which is further supplemented by the boxplot to give an idea regarding the descriptive statistics (median and inter-quartile range). The raincloud plot was created using DATAtab Team (2024). DATAtab: Online Statistics Calculator. DATAtab e.U. Graz, Austria. URL https://datatab.net.

A two-factor analysis of variance with measurement repetition was performed to test whether there was a significant difference between the groups of the first factor, "Age and Grade" (repeated measures), with respect to the dependent variable. There is a significant difference between the groups of the second factor, “Level of Knowledge and Attitude,” in relation to the dependent variable. There is an interaction between the two factors "age and grade" and “level of knowledge and attitude” in relation to the dependent variable. The two-factor analysis of variance with repeated measures showed that there is a significant difference between the groups of the first factor, "Age and Grade," in relation to the dependent variable, p = 0.001; a significant difference between the groups of the second factor, ”Level of Knowledge and Attitude,” in relation to the dependent variable, p =.001; and no interaction between the two variables, “Level of Knowledge and Attitude” and "Age and Grade," in relation to the dependent variable, p = 0.15. The data visualisation, taking into consideration age, grade, and categories of level of knowledge and attitude, is depicted in the form of a raincloud plot (Figure 3).

## Discussion

In this study on the knowledge and attitude regarding HIV/AIDS among the adolescent population, the knowledge and attitude were found to be good in only 165 (52.5%) of study participants. The majority of 178 (56.7%) of the participants mentioned their source of knowledge as being from school or college. The majority of participants displayed good knowledge about HIV/AIDS. Specifically, 250 (79.6%) students knew that unprotected sex with an infected partner could transmit HIV, and 202 (64.3%) were aware of the risk of mother-to-child transmission. Attitudes towards individuals living with HIV/AIDS were also largely positive. A significant portion of the participants, 192 (61.1%) agreed that HIV-infected individuals should be allowed to study in the same school, and 183 (58.3%) believed that such individuals should live without isolation. The study found significant associations between knowledge, attitude levels, and factors such as age, grade, and religion. Additionally, the logistic regression analysis indicated significant influences of age, grade, and religion on good knowledge and attitude, emphasising targeted educational interventions. The primary source of knowledge about HIV/AIDS for the majority of the participants, 178 (56.7%), was their educational institution, emphasising the role of educational institutions in providing health-related information.

Age, education, socioeconomic class, area, availability of mass media, and other factors all have an impact on a population's HIV/AIDS knowledge [12,13]. As a developing country, there will be significant differences in all of these elements from region to region in India [14]. In the present study, good knowledge and attitude were found to be more prevalent in male participants, 100 (32%), as compared to female participants, 65 (21%), but no statistical significance was found. In a similar study done in Gujarat, a high level of awareness was found in male participants as compared to female participants, and it was not statistically significant [15]. Good knowledge and attitude were found to be more prevalent, with 111 (35%) in the age group of 17 and below. Similar findings were obtained in the study done in Gujarat by Kariya et al., where adolescents in the age group of 17 performed the highest in the questionnaire [15]. On the contrary, most research supports the notion that as an adolescent's age increases, so does his or her knowledge. The UDAYA (Understanding the Lives of Adolescents and Young Adults) project, a research undertaking by Srivastava et al. on adolescents' knowledge and attitudes, also supports the age hypothesis [16].

The majority, 202 (64.3%) of the study participants, agreed that there is a risk of mother-to-child transmission if the mother is infected with HIV. The findings that were obtained in a study done in Mizoram by Pachuau et al. were significantly higher, which stated that 2570 (84.2%) reported knowing about mother-to-child transmission [17]. Similar results were obtained in a study done in Kamrup (Metro) by Pegu and Gaur, where 213 (74.2%) reported knowing that HIV can be transmitted from mother to child [18]. The majority, 250 (79.6%) of the participants, agreed with the fact that a person can get infected with HIV/AIDS by having unprotected sex with their infected partner. In a study done in Kamrup (Metro) by Pegu and Gaur, 273 (95.1%) of respondents knew about the sexual mode of transmission of HIV/AIDS, which is remarkably higher [18]. 

In the present study, the majority, 202 (64.3%) of the study participants, agreed that a person can get HIV if they get HIV-contaminated blood. A study done in Kamrup (Metro) by Pegu and Gaur reported that 235 (81.8%) knew about HIV transmission through contaminated blood [18]. The majority, 185 (58.9%) of the study participants, agreed that using the same syringe with an HIV-infected person can transmit HIV/AIDS. In the study done in Kamrup (Metro) by Pegu and Gaur, 198 (69%) reported knowing about the transmission by contaminated syringes [18]. In the present study, the majority, 183 (58.3%) of the study participants, agreed that it is acceptable for an HIV-infected person to live freely without isolation, showing a positive attitude. Similar findings were found in the study done in Kamrup (Metro) by Pegu and Gaur, where 191 (66.5%) had a positive attitude that HIV/AIDS patients should not be isolated [18].

The Adolescent Education Programme's efficacy is evidenced by participants' high levels of knowledge and overall favourable attitudes. This shows that continuing and expanding the implementation of such programmes could help raise HIV/AIDS knowledge and reduce stigma. Given the differences in knowledge and attitudes based on sociodemographic characteristics, personalised interventions that consider age, educational level, and cultural contexts may be more effective. 

The study design is robust, and the study emphasises the school-based programme. It focuses on knowledge as well as attitude, which is crucial in reducing high-risk behaviours and stigma as well as discrimination against HIV/AIDS. As important sociodemographic factors were analysed regarding their effects on knowledge and attitudes regarding HIV/AIDS, it offers future prospects for targeted intervention in those areas. It was also noticed that a few schools showed hesitancy in regard to the subject matter, fearing that the guardians of the students might object to the study; hence, there is a prospect of future interventions in those particular educational institutions.

There are a few limitations to the study. The study was limited to a specific geographic area (Kamrup Metro) and a relatively small sample size, which may not be representative of all adolescents in Assam or other regions of India. The use of self-reported data to assess knowledge and attitudes may induce bias, as participants may produce socially desired responses. One important factor regarding comprehensive knowledge and attitude towards HIV/AIDS is the proper implementation of the adolescent education programme by competent teachers who are trained for that, but there may be some institutions where there is a dearth of competent teachers, which can reflect in the study results with the poor performance of the students.

## Conclusions

The study highlights the significant role of education in improving knowledge regarding HIV/AIDS as well as developing a positive attitude towards HIV/AIDS among adolescents. Repeated efforts in school-based programmes and targeted interventions based on influential sociodemographic variables can pave the path for adolescents, reducing high-risk behaviour and stigma associated with HIV/AIDS. The successful execution of India's Adolescent Education Programme (AEP) has several obstacles, the most prominent of which are inadequate infrastructure, a shortage of skilled teachers, and bureaucratic inefficiencies. The goals, as stated in the Adolescent Education Programme, emphasise teaching adolescent students life skills and cultivating positive attitudes regarding HIV/AIDS, but there are still significant actions left to be undertaken by the Government of India, such as strengthening infrastructure, training teachers, and engaging more manpower.
